# Lower resting and total energy expenditure in postmenopausal compared with premenopausal women matched for abdominal obesity

**DOI:** 10.1017/jns.2013.38

**Published:** 2014-02-13

**Authors:** Leanne Hodson, Karin Harnden, Rajarshi Banerjee, Belen Real, Kyriakoula Marinou, Fredrik Karpe, Barbara A. Fielding

**Affiliations:** 1 Oxford Centre for Diabetes, Endocrinology and Metabolism, Churchill Hospital, Oxford OX3 7LE, UK; 2 Oxford Centre for Clinical Magnetic Resonance Research, Division of Cardiovascular Medicine, John Radcliffe Hospital, Oxford OX3 9DU, UK; 3 Department of Experimental Physiology, Athens University School of Medicine, Greece; 4 National Institute for Health Research, Oxford Biomedical Research Centre, Oxford University Hospital Trusts, Oxford OX3 7LJ, UK; 5 Faculty of Health and Medical Sciences, University of Surrey, Guildford GU2 7WG, UK

**Keywords:** Diet, Body composition, Bone health, Fatty acids, AEE, activity energy expenditure, AT, adipose tissue, BMD, bone mineral density, FSH, follicle-stimulating hormone, MET, metabolic equivalent, TEE, total energy expenditure

## Abstract

The menopause is accompanied by increased risk of obesity, altered body fat distribution and decreased skeletal muscle mass. The resulting decrease in RMR should be accompanied by a compensatory change in energy balance to avoid weight gain. We aimed to investigate habitual energy intake and expenditure in pre- and postmenopausal women matched for abdominal obesity. We recruited fifty-one healthy Caucasian women, BMI > 18·5 and <35 kg/m^2^, aged 35–45 years (premenopausal, *n* 26) and 55–65 years (postmenopausal, *n* 25). Energy intake was measured using 3 d diet diaries and dietary fat quality assessed using adipose tissue fatty acid biomarkers. RMR was measured using indirect calorimetry, and total energy expenditure (TEE) and activity energy expenditure using a combined accelerometer and heart rate monitor. Postmenopausal women had lower RMR and TEE and spent significantly less time undertaking moderate exercise than premenopausal women. Postmenopausal women had a tendency for a lower energy intake, and a similar macronutrient intake but a significantly lower adipose tissue *n*-6:*n*-3 ratio (24·6 (se 1·6) *v*. 37·7 (se 3·1); *P* < 0·001). The main lifestyle determinant of bone mineral density (which was significantly lower in postmenopausal women) was TEE for premenopausal women, and dietary *n*-6:*n*-3 ratio for postmenopausal women. The present results suggest that weight maintenance is achieved in the post- compared with premenopausal status through a combination of reduced energy intake and reduced TEE in a regimen that compromises micronutrient intake and has a negative impact on lean tissue mass. However, lower *n*-6:*n*-3 fatty acid intake in postmenopausal women is associated with greater bone mineral density.

The combined effect of ageing and the menopause leads to a sharp increase in the risk of adverse clinical outcomes such as incidence and mortality rates for CHD^(^^1^^)^ and bone fracture^(^^2^^)^ in women. In fact, these two clinical outcomes are related; low bone mineral density (BMD), a risk factor for osteoporosis, has been associated with cardiovascular endpoints^(^^3^^)^. Fat mass and lean mass have both been shown to be significantly associated with BMD as reviewed^(^^4^^)^ and lower abdominal fat mass has recently been shown to be associated with higher risk of bone fracture, independently of BMD^(^^5^^)^. However, the latter study did not distinguish between intra-abdominal and subcutaneous fat. Exercise is also strongly associated with BMD, and exercise therapy has been shown to increase BMD in randomised controlled trials^(^^6^^)^.

The menopause is associated with small but unfavourable changes in body fat distribution. Lovejoy *et al.*^(^^7^^)^ followed 156 middle-aged, healthy, premenopausal women and divided them according to menopausal status (pre-, peri- and postmenopausal) after 4 years. All women gained subcutaneous adipose tissue (AT) but only those who became postmenopausal had a significant increase in visceral AT, determined by a computed tomography scan at the level of the L4–L5 vertebrae. Larger studies show an increased risk of obesity in mid-life. The Health Survey for England 2011^(^^8^^)^ showed that 61 % of women aged 35 to 44 years were overweight or obese compared with 69 % women aged 55 to 64 years. Sowers *et al.*^(^^9^^)^ showed that in 800 women from the Study of Women's Health Across the Nation (SWAN) who were followed over 6 years through the menopausal period, the average increase in fat was 3·4 kg and waist circumference was 5·7 cm. These changes were not associated with menopausal stage but with a calculation of ‘ovarian ageing’. Follicle-stimulating hormone (FSH) levels were found to positively correlate with the change in fat mass. In agreement, women in the Healthy Women Study gained 0·7 kg/year in their 40s and 50s, which was not dependent on menopause status^(^^10^^)^.

With increasing obesity, there is an increase in cardiovascular risk factors. In a study including 275 women aged 22–71 years, insulin resistance, systolic blood pressure, low plasma HDL-cholesterol and TAG were found to increase with BMI^(^^11^^)^. Menopausal status was not considered in the analysis. However, it has been shown that plasma total cholesterol, LDL-cholesterol and apoB specifically increase to a greater extent than would be expected by ageing during the menopause^(^^12^^)^. A healthy lifestyle to reduce risk of CVD is therefore particularly important in postmenopausal women. Exercise is beneficial to health and a recent study has shown that habitual running and walking are associated with similar risk reductions of hypercholesterolaemia and hypertension^(^^13^^)^. Although women of menopausal age were included in the cohort, menopausal status was not reported.

There is a decrease in lean mass in women after the menopause which is related to years since menopause rather than age^(^^14^^)^, and a decrease in lean mass is associated with a decrease in RMR^(^^15^^)^. Moreover, it has been shown that postmenopausal women have a lower fat oxidation and energy expenditure during exercise than premenopausal women^(^^16^^)^, further contributing to a lower capacity for substrate utilisation by skeletal muscle after the menopause. Lovejoy *et al.*^(^^7^^)^ also showed that both free energy expenditure (and activity energy expenditure; AEE) and sleeping energy expenditure declined over time in mid-life women (the decrease in sleeping energy expenditure was 1·5 times greater in women who became postmenopausal over the 4-year follow-up period). If both RMR and AEE are reduced and there is no equivalent reduction in energy intake, then an increase in BMI along with increased health risks will be inevitable. In a subset of the study mentioned above by Lovejoy *et al.*^(^^7^^)^, a significant decrease in 24 h energy expenditure and lower spontaneous physical activity was found in women who had become postmenopausal. It is not known which has the greater effect on weight gain during and post-menopause: the reduction of total energy expenditure (TEE) or change in energy intake. Some studies have found that macro- and micronutrient intake is unchanged in the early years following the menopause (less than or equal to 6 years) with a small increase or no change in weight^(^^7^^,^^17^^)^. Waist circumference is considered to be a risk factor for CVD^(^^18^^)^ and this has been adopted as a simple measure of health risk^(^^19^^)^. We aimed to determine lifestyle factors that are associated with the maintenance of waist circumference in the transition to postmenopausal status in healthy women. Therefore, in a cross-sectional study, we compared energy intake *v.* energy expenditure in a well-characterised cohort of pre- and postmenopausal women, matched for abdominal obesity. We used an Actiheart^®^ combined accelerometer and heart rate monitor, as well as indirect calorimetry to accurately assess habitual energy expenditure and a combination of 3 d diet diaries and fatty acid biomarkers to assess food intake. We then assessed how these variables made an impact on bone health, determined by whole-body BMD.

## Methods

### Subjects

A total of 51 volunteers were recruited by advertisement and from the Oxford BioBank^(^^20^^)^. All volunteers were metabolically healthy and not taking medication known to affect lipid or glucose metabolism, including any supplements containing *n*-3 PUFA or *n*-6 fatty acids. The present study was part of a larger study investigating metabolism in pre- and postmenopausal women. It was conducted according to the guidelines laid down in the Declaration of Helsinki and all procedures involving human subjects were approved by the Oxfordshire Clinical Research Ethics Committee. Written informed consent was obtained from all subjects.

All volunteers were Caucasian women, with a BMI > 18·5 and <35 kg/m^2^, aged between 35 and 45 years (premenopausal) and 55 and 65 years (postmenopausal) and who reported being weight stable for a period of 2 months before the study. Subjects were excluded if they were taking oral contraceptives or hormone replacement therapy. We recruited twenty-six pre- and twenty-five postmenopausal women. Premenopausal was defined as having regular menses over the past 12 months and blood FSH < 30 IU/l, whilst postmenopausal status was defined as absence of menses for at least 12 months and blood FSH > 30 IU/l. The pre- and postmenopausal groups were matched for BMI and waist circumference. The participants' characteristics are given in Table 1.

**Table 1.** Participant characteristics (Mean values with their standard errors)

	Premenopausal (*n* 26)		Postmenopausal (*n* 25)		
	Mean	se	Mean	se	*P*
Age (years)	41	0·6	58	0·5	<0·001
Systolic blood pressure (mm/Hg)	113	2	125	3	0·002
Diastolic blood pressure (mm/Hg)	75	2	75	2	0·751
Height (cm)	166	1·2	163	1·4	0·095
Weight (kg)	69·2	2·3	66·0	1·6	0·277
BMI (kg/m^2^)	25·0	0·7	24·8	0·5	0·811
Waist (cm)	83	2	82	2	0·653
Waist:hip ratio	0·84	0·01	0·84	0·01	0·589
Fat (kg)	23·5	1·5	23·5	0·9	0·983
Fat (% body weight)	33·2	1·2	35·4	0·7	0·135
Lean mass (kg)	42·3	1·0	39·3	0·9	0·029
Lean (% body weight)	61·9	1·1	59·7	0·9	0·139
Subcutaneous fat (cm^2^)	226	18	240	15	0·572
Visceral fat (cm^2^)	41·2	4·7	54·3	6·4	0·107
Bone mineral content (kg)	2·57	0·05	2·20	0·06	<0·001
Bone mineral density (g/cm^2^)	1·19	0·01	1·05	0·02	<0·001
Liver fat (%)	1·7	0·5	1·6	0·3	0·884

### Food diaries

All subjects were given a food diary at the screening visit and asked to complete three non-consecutive days (1 weekend day and 2 week days) before the study day. The food diaries used were provided by the European Prospective Investigation of Cancer and Nutrition (EPIC), Cambridge and had been previously validated^(^^21^^)^. The diaries contained detailed descriptions of the information required, along with photographs to help subjects quantify the amount of specific foods. The energy and nutrient composition of the diets was calculated using the Microdiet 2 program (Downlee Systems Ltd).

### Body composition

A dual-energy X-ray absorptiometry (GE Health Care Lunar iDexa, with software version 14.1) scan was carried out to measure lean body mass, fat body mass, and bone mineral content and density. Height was measured to the nearest 0·1 cm without shoes, and weight to the nearest 0·1 kg in light clothing. Waist was measured using the mid-point between the lower rib and ileac crest. Hip circumference was the widest point of the buttocks.

### Subcutaneous, visceral and hepatic fat

Intra-hepatic, visceral and subcutaneous fat was measured at the Oxford Centre for Clinical Magnetic Resonance Research after an overnight fast and within 2 weeks of the study day, using a 3·0 Tesla magnetic resonance (MR) scanner (Siemens). Hepatic fat content was determined using MR spectroscopy; spectra were acquired from an 8 cm^3^ voxel positioned in the right lobe of the liver, avoiding blood vessels and the biliary tract. Peaks for water and lipid resonances were quantified from the spectra, and liver fat expressed as a percentage of the hepatic water content. For visceral and subcutaneous fat, images of the abdomen at the level of the L4–L5 vertebrae were acquired using MRI.

### Adipose tissue analysis

A subset of women consented to AT biopsies (*n* 19 and *n* 23 for pre- and postmenopausal women, respectively). Gluteal subcutaneous AT samples were taken by needle biopsy^(^^22^^)^ from the upper buttock area. AT fatty acid composition was measured on the lipid layers generated by RNA extraction^(^^23^^)^. It was not possible to measure the content of 15 : 0, which was used as an internal standard.

### RMR

RMR was measured in the overnight fasting state using an open-circuit indirect calorimeter, the Gas Exchange Measurement (GEM) analyser (GEMNutrition Ltd). Over an 8 h period, two fasting measurements, each lasting 23 min, were made to assess RMR, and whole-body respiratory quotient.

### Measurement of total and activity energy expenditure

AEE was measured using an Actiheart monitor (CamnTech) using the RMR measured by indirect calorimetry. The Actiheart records physical activity and heart rate and uses a system of branched modelling to calculate AEE. The lightweight, waterproof monitor clips on to electrocardiogram electrode pads attached to the upper chest. After performing a signal test the subjects were asked to wear the monitor for 7 full days. Once returned, the information stored by the monitor was downloaded. TEE is calculated as the RMR plus measured AEE and calculated diet-induced thermogenesis. Energy expenditure is also expressed as an average intensity in terms of metabolic equivalents (MET), which represent multiples of RMR. We calculated the time spent carrying out activities at levels of MET corresponding to light (<3), moderate (3–6) and vigorous (>6) intensity exercise.

### Data analysis

Using data from a previous study that measured sleeping energy expenditure in pre- and postmenopausal women^(^^7^^)^, the number of subjects in pre- and postmenopausal groups (unpaired) to detect a 10 % difference with power of 0·80 at α of 0·05 is eighteen. Therefore the present study with twenty-six and twenty-five pre- and postmenopausal women was adequately powered to find differences in resting RMR. Data analysis was performed using SPSS Statistics 19 (IBM). Data are presented as mean values with their standard errors and groups were compared using unpaired *t* tests, using log-transformed data where appropriate. Correlations were performed using Pearson's correlation coefficient. The 10-year CVD risk was calculated using the general CVD calculator from the Framingham Heart Study^(^^24^^)^.

## Results

### Participant characteristics

Postmenopausal women were significantly older and had the same waist circumference according to our recruitment criteria (Table 1). Groups were not significantly different for BMI, fat mass and all measures of adiposity, but postmenopausal women had less lean mass compared with premenopausal women (*P* = 0·029), although there was no significant difference when expressed as percentage of body weight (data not shown). Systolic blood pressure was significantly higher in postmenopausal women (*P* = 0·002). Postmenopausal women had significantly lower bone mineral content and BMD (both *P* < 0·001) than premenopausal women.

### Fasting biochemical parameters

Plasma FSH was significantly higher in postmenopausal women (range 47–125 IU/l), with all women well above the cut-off of 30 IU/l, compared with premenopausal women (range 3–21 IU/l) (*P* < 0·001) (Table 2). Plasma total cholesterol and LDL-cholesterol were significantly higher in postmenopausal women but HDL-cholesterol, TAG, insulin and glucose were similar (Table 2).

**Table 2.** Fasting plasma biochemical profiles and estimated 10-year CVD risk (Mean values with their standard errors)

	Premenopausal (*n* 26)		Postmenopausal (*n* 25)		
	Mean	se	Mean	se	*P*
Follicle-stimulating hormone (IU/l)	7·2	0·8	84·3	4·3	<0·001
Total cholesterol (mmol/l)	4·92	0·13	5·91	0·17	<0·001
HDL-cholesterol (mmol/l)	1·66	0·07	1·65	0·07	0·957
LDL-cholesterol, calculated (mmol/l)	2·87	0·13	3·70	0·17	<0·001
TAG (mmol/l)	0·89	0·15	0·93	0·08	0·848
Glucose (mmol/l)	4·94	0·07	5·02	0·09	0·434
Insulin (pmol/l)	79·9	7·6	72·9	2·8	0·363
10-year Framingham risk score (%)	1·83	0·2	6·50	0·5	<0·001

### Dietary intake

There was a tendency for postmenopausal women to have lower total energy intake (mean difference 819 kJ) (Table 3). There were no differences in macronutrient intakes between the groups. When expressed as absolute intake (g/d) there was a tendency for premenopausal women to report a higher alcohol intake than postmenopausal women (Table 3); this difference was not seen when the data were expressed as a percentage of total energy intake (data not shown). There were no differences in NSP, vitamin or mineral intakes between the groups (Table 3).

**Table 3.** Average daily total energy and nutrient intake collected from 3 d diet diaries (Mean values with their standard errors)

	Premenopausal (*n* 26)		Postmenopausal (*n* 25)		
	Mean	se	Mean	se	*P*
Total energy (kJ)	8134	364	7315	61	0·073
Carbohydrate					
g	227	10	217	8	0·478
% Dietary energy	44	1	47	1	NS
Total sugars					
g	110	7	106	4	0·652
% Dietary energy	22	1	23	1	NS
Starch					
g	112	6	108	6	0·644
% Dietary energy	22	1	23	1	NS
Protein					
g	76	3	68	3	0·092
% Dietary energy	16	1	16	1	NS
Fat					
g	77	6	67	4	0·172
% Dietary energy	35	1	34	1	NS
Alcohol					
g	15	3	8	2	0·08
% Dietary energy	5	1	4	1	NS
NSP (g)	16	1	18	1	0·228
Ca (mg)	930	41	873	55	0·405
Mg (mg)	319	18	293	14	0·263
P (mg)	1360	55	1257	50	0·174
Fe (mg)	13	1	11	1	0·216
Vitamin D (µg)	2·8	0·3	2·8	0·5	0·915

### Dietary biomarkers

AT fatty acid composition was used as a biomarker marker of 16 : 0, 18 : 2*n*-6, 20 : 4*n*-6, 20 : 5*n*-3 and 22 : 6*n*-3 intake^(^^25^^)^. The proportion of 18 : 1*n*-9 in AT was also measured but with the proviso that this fatty acid is not reflective of diet^(^^25^^)^. The proportion of 17 : 0 was measured as a measure of dairy intake^(^^26^^)^. The proportions of AT 16 : 0, 17 : 0 and 18 : 1*n*-9 were similar in pre- and postmenopausal women but the proportions of 18 : 2*n*-6, 20 : 4*n*-6, 20 : 5*n*-3 and 22 : 6*n*-3 were significantly higher in postmenopausal women (Table 4). The ratio of AT *n*-6:*n*-3 was lower in postmenopausal women (*P* < 0·001). The proportions of 18 : 2*n*-6 and 20 : 4*n*-6 were positively correlated (*r* 0·303; *P* = 0·036). Moreover, when split into pre- and postmenopausal groups the correlation between 18 : 2*n*-6 and 20 : 4*n*-6 was significant in pre- (*r* 0·46; *P* = 0·049) but not postmenopausal women.

**Table 4.** Biomarkers of fatty acid intake: gluteal subcutaneous adipose tissue fatty acid composition (mol/100 mol)* (Mean values with their standard errors)

	Premenopausal (*n* 19)		Postmenopausal (*n* 23)		
Fatty acid	Mean	se	Mean	se	*P*
16 : 0	21·0	0·3	20·4	0·4	0·289
17 : 0	0·26	0·02	0·26	0·02	0·953
18 : 1*n*-9	50·1	0·3	49·2	0·42	0·111
18 : 2*n*-6	11·8	0·3	13·1	0·5	0·044
20 : 4*n*-6	0·32	0·02	0·35	0·02	0·127
20 : 5*n*-3	0·071	0·007	0·11	0·01	<0·001
22 : 6*n*-3	0·12	0·016	0·21	0·01	<0·001
Ratio *n*-6:*n*-3	37·7	3·1	24·6	1·6	<0·001

* Nineteen of twenty-six and twenty-three of twenty-five pre- and postmenopausal women, respectively, consented to give adipose tissue biopsies.

### Energy expenditure

Postmenopausal women had significantly lower RMR compared with premenopausal women, which was not different when corrected for lean mass (data not shown). Postmenopausal women had a significantly lower TEE (*P* = 0·029), with a tendency for a lower AEE (Table 5). The activity counts were significantly lower in postmenopausal women compared with premenopausal women (*P* = 0·012). Therefore we looked at time spent exercising at various intensities.

**Table 5.** Energy expenditure (Mean values with their standard errors)

	Premenopausal (*n* 26)		Postmenopausal (*n* 25)		
	Mean	se	Mean	se	*P*
RMR (kJ/d)	6215	170	5722	131	0·029
TEE (kJ/d)	12695	660	10876	464	0·029
Activity counts (per min)	45	5	31	2	0·012
MET (0–3 min)	1245	26·9	1296	17·8	0·067
MET (3–6 min)	183	25·3	140	17·8	0·03
MET (>6 min)	11·8	3·0	4·94	1·27	0·12
RQ	0·79	0·01	0·77	0·01	0·434
AEE (kJ/d)	5221	506	4076	376	0·076

TEE, total energy expenditure; MET, metabolic equivalents of task; RQ, respiratory quotient; AEE, activity energy expenditure.

Premenopausal women spent a significantly greater proportion of the day performing moderate-intensity exercise (MET 3–6) than postmenopausal women (*P* = 0·03). Postmenopausal women had a tendency to perform slightly more light-intensity activities (MET 0–3) (Table 5). There was no difference in whole-body respiratory quotient during fasting, showing no difference in substrate utilisation in the fasting state. None of the measures of energy expenditure was correlated with plasma total or LDL-cholesterol.

### Determinants of bone mineral density

Weight and lean mass were positively associated with BMD in the whole cohort (*r* 0·40, *P* = 0·016; and *r* 0·49, *P* = 0·0002, respectively) but this was due to significant associations in pre- (*r* 0·46, *P* = 0·017; and *r* 0·53, *P* = 0·005, respectively) but not postmenopausal women. TEE was correlated with lean mass in pre- (*r* 0·49; *P* = 0·011) and postmenopausal women (*r* 0·58; *P* = 0·003). The AT *n*-6:*n*-3 ratio was significantly negatively correlated with BMD in post- (*r* –0·44; *P* = 0·044) but not premenopausal women (*r* 0·21; *P* = 0·4). We carried out linear regression in order to determine which of the two lifestyle variables most related to BMD, TEE or dietary *n*-6:*n*-3 ratio (input as AT *n*-6:*n*-3). For premenopausal women, TEE was an independent predictor of BMD (β = 0·56; *P* = 0·013), whereas for postmenopausal women, it was dietary *n*-6:*n*-3 (β = –0·44; *P* = 0·044).

## Discussion

Due to a combination of hormonal changes and ageing, often exacerbated by weight gain, postmenopausal women have increased CVD risk and poorer bone health than premenopausal women. Since the avoidance of weight gain in the postmenopausal transition would be considered beneficial, we identified lifestyle factors that were associated with maintenance of adiposity in the present cross-sectional study. With a novel combination of objective measurements of energy expenditure and dietary intake, we were also able to identify different lifestyle factors that were associated with BMD in post- compared with premenopausal women.

Postmenopausal women achieved energy balance by having a significantly lower TEE (including significantly lower RMR and a tendency for a lower AEE), and a tendency for lower (dietary) energy intake, compared with the premenopausal women. Moreover, the postmenopausal women spent a significantly lower proportion of the day performing moderate-intensity exercise than premenopausal women. Using biomarkers of fatty acid intake we found that postmenopausal women consumed significantly higher proportions of 18 : 2*n*-6 and *n*-3 PUFA, with a lower *n*-6:*n*-3 ratio than premenopausal women. BMD was significantly lower in postmenopausal women, and lower levels of habitual exercise may have contributed to the poorer bone health of postmenopausal women. However, there is some evidence to suggest that the BMD in postmenopausal women was related to the *n*-6:*n*-3 ratio in the diet.

We found no significant differences in measures of adiposity, body fat distribution or liver fat between pre- and postmenopausal women matched for waist. This included BMI, hip, waist:hip ratio and android/gynoid fat (data not shown). This was surprising, as we expected to find a change in body fat distribution based on many previous studies, as recently reviewed^(^^27^^)^. However, differences in adiposity and body fat distribution between pre- and postmenopausal women are not reported in all studies. Our data are in agreement with those of Abildgaard *et al.*^(^^16^^)^, who found no difference in weight, fat mass, trunk fat mass and visceral fat mass in non-obese pre- and postmenopausal women in a cross-sectional study. We found postmenopausal women had a significantly lower mean lean body mass (3 kg) than premenopausal women, which was similar to the value of 4·3 kg found by Abildgaard *et al.*^(^^16^^)^. Not all studies find a difference in lean body mass; Lovejoy *et al.*^(^^7^^)^ reported no change in lean mass when women transitioned to the menopause in a longitudinal study, despite following a cohort of women who were <3 years menopausal, which was similar to the difference in age in the cross-sectional study of Abildgaard *et al.*^(^^16^^)^. It has been suggested that liver fat accumulation increases after the menopause^(^^28^^,^^29^^)^ but we found no evidence of this in our cohort which included mainly lean and overweight individuals.

A lower lean mass in postmenopausal women is associated with a lower RMR and we found a significantly lower value of 493 kJ, coupled with a significantly lower TEE (1819 kJ) in our cohort of post- compared with premenopausal women. Therefore, because BMI was similar in the two groups, we expected to find lower reported energy intake in the postmenopausal group but we only found a tendency for this (819 kJ lower per d). However, this difference, although not significant, could be very important. Without this lower intake, it can be estimated that, in theory, a weight gain of 8·8 kg/year^(^^30^^)^ would ensue, further increasing CVD risk. Estimates of energy intake from food diaries are well reported to be underestimated and the 3 d diet diaries used in the present study may have been insufficient; it is possible that if we had requested 5 or 7 d we may have seen a significant difference in the energy intake. Under-reporting of dietary intake has been defined as the ratio of EI to (calculated) BMR < 1·05^(^^31^^)^ and in the present study, only three women fell into this category. If a more stringent cut-off of <1·35 is used^(^^32^^)^, which probably includes acceptable reporters^(^^31^^)^, fifteen (58 %) pre- and fifteen (56 %) postmenopausal women would be classified as ‘under-reporters’. This is not surprising, as studies using doubly labelled water have shown that with increasing BMI there is greater under-reporting and also even lean, healthy, very informed dietitians under-report by 16 %^(^^33^^,^^34^^)^. However, the ratio of EI:BMR was not significantly different between pre- and postmenopausal women in the present study (data not shown), suggesting no difference in under-reporting between the two groups.

As all the women in the present study reported to be weight stable we expected that the energy intakes would be nearer to the TEE. We used a combined accelerometer and heart rate monitor to measure activity and TEE in the present study. This approach has been used in many studies in recent years to provide valuable information on habitual activity in free-living individuals^(^^35^^,^^36^^)^. The technique is non-invasive and relatively cheap compared with other methodologies used in human research, and is more objective than diaries and questionnaires^(^^37^^)^. However, we cannot exclude the possibility that the participants in the present study exercised more than usual because they were not blinded to the measurement. Postmenopausal women spent less time exercising at moderate intensity than premenopausal women, which is in agreement with previous studies. Lovejoy *et al.*^(^^7^^)^ noted a non-significant decrease in physical activity, as measured by triaxial accelerometer, as women transitioned from pre- to post-menopause. However, in an experimental setting (whole-room calorimetry) the authors found significant decreases in 24 h energy expenditure and spontaneous physical activity with age in women in those that transitioned to being postmenopausal but also in those who stayed premenopausal. This is in accordance with a marked decrease in activity with age (uncorrected for BMI)^(^^38^^)^.

Postmenopausal women had markedly higher proportions of 18 : 2*n*-6 (1·3 mol/100 mol) and small but significantly higher *n*-3 PUFA (<0·2 mol/100 mol) in AT but no significant difference in 16 : 0, 18 : 1*n*-9 or 20 : 4*n*-6. Previous studies have reported limited associations between AT 16 : 0 or 18 : 1*n*-9 and dietary intake but a consistent finding is the robust associations between the abundance of AT and dietary intakes of 18 : 2*n*-6 and *n*-3 PUFA^(^^25^^)^. The proportion of 20 : 4*n*-6 in AT has been shown to be related to intake in an animal model but this was not found in human subjects^(^^39^^)^. We found no difference in AT 20 : 4*n*-6 between pre- and postmenopausal women. Our findings indicate that about 20 % of the variance in AT 20 : 4*n*-6 is explained by the proportion of 18 : 2*n*-6, suggesting that endogenous synthesis of 20 : 4*n*-6 might be more important in premenopausal women. A case–control study found that gluteal AT 20 : 4*n*-6 was positively associated with the risk of incidence of myocardial infarction but was not associated with 18 : 2*n*-6. The biological relevance of the association between AT 20 : 4*n*-6 remains to be elucidated^(^^39^^)^. Moreover, the effect of menopausal status on the findings of the study was not investigated although menopausal status of the women in the study was considered as a potential confounder. AT *n*-3 PUFA in our cohort were surprisingly comparable with data we have previously collated across many countries^(^^25^^)^, despite a low intake of oily fish in the UK population^(^^40^^)^. Overall, although there was no difference in the total fat intakes, biomarkers of specific dietary fatty acids indicated that the composition of dietary fat differed between the groups.

We found a significant correlation between AT *n*-6:*n*-3 fatty acids and BMD in postmenopausal women. Long-chain *n*-3 PUFA have been shown to be beneficial for bone health, as recently reviewed^(^^41^^)^. Potential mechanisms for their action are complicated and are thought to include reduction in PGE2 synthesis, with suppression of inflammatory cytokines. Conversely, the *n*-6 fatty acid arachidonic acid may have an opposing effect. Thus the ratio of *n*-3:*n*-6 fatty acids has been implicated as being important as reviewed^(^^42^^)^ and this is supported by animal studies; however, human studies have not all been in accordance^(^^43^^,^^44^^)^. We did not find an association between AT *n*-6:*n*-3 fatty acids and BMD in premenopausal women; this may have been due to the low intake of *n*-3 fatty acids in that group. Therefore, further work is required to establish the relationship between *n*-6:*n*-3 fatty acids and bone health in women. It is important to point out that dietary 18 : 2*n*-6 has health benefits; for example, substitution of 18 : 2*n*-6 for saturated fat is known to reduce plasma cholesterol^(^^45^^)^. However, *n*-6 and *n*-3 fatty acids have opposing effects; both are ligands/modulators for nuclear receptors which control various genes involved in inflammatory signalling and lipid metabolism; thus the ratio of *n*-6:*n*-3 within the cell may strongly influence its function^(^^46^^)^.

Weight and lean mass were positively associated with BMD in pre- but not postmenopausal women. This is in agreement with previous work that showed a significant association between lean mass and BMD in pre- and early peri-menopausal women that was stronger than the association between fat mass and BMD^(^^47^^)^. Regarding postmenopausal women, some previous studies have shown fat mass and not lean mass was a significant determinant of BMD^(^^48^^–^^52^^)^, in agreement with our findings, but this was not found in other studies^(^^47^^,^^53^^–^^55^^)^. A study looking at factors determining BMD in pre- and postmenopausal women in Japan found that lean mass was the most significant determinant of lumbar and total BMD in premenopausal women whereas fat mass was the most important predictor in postmenopausal women aged 45–83 years^(^^56^^)^. We did not find an association between fat mass and BMD in postmenopausal women in the present study. This may be due to smaller numbers, smaller BMI range or a smaller age range in the present study (55–65 years for postmenopausal women). Exercise is also a confounder; it has been reported that BMD is only associated with fat mass in sedentary women^(^^57^^)^. We found that moderate-intensity exercise was correlated with lean mass and BMD in postmenopausal women, but postmenopausal women undertook significantly less moderate exercise than premenopausal women.

At present the current RDA for Ca in the UK for men and women aged 19+ years is 700 mg/d^(^^58^^)^. However, because of increased risk of osteoporosis after the menopause, higher Ca intake is sometimes recommended for postmenopausal women (for example, The British Dietetic Association recommends 700 mg/d for women 19+ years and 1200 mg/d for postmenopausal women^(^^59^^)^). The recommended Ca intakes based on Western European, American and Canadian data are 1000 mg/d for females 19 years until menopause and then 1300 mg/d post-menopause^(^^60^^)^. We found no significant difference in Ca intake for pre- and postmenopausal women; in agreement with this, we found no difference for AT 17 : 0 (a marker of dairy intake which is a major source of Ca in the diet). The mean intake of Ca of the postmenopausal group in the present study falls short of some of these recommendations (by over 400 mg/d). Postmenopausal women should be aware that if total energy intake is decreased, micronutrients such as Ca may decrease in parallel unless efforts are made to maintain a healthy, well-balanced diet.

Plasma total and LDL-cholesterol concentrations were significantly higher in the postmenopausal women, despite no difference in weight compared with the premenopausal women, as found previously^(^^12^^)^. The higher plasma total cholesterol concentrations together with higher systolic blood pressure in the postmenopausal group are the major modifiable determinants of 10-year CVD risk using the Framingham risk equation (based on lipids), which was on average 3·6-fold higher in the postmenopausal group. This risk equation does not take into account the lower exercise in the postmenopausal group which could further increase plasma cholesterol and blood pressure and exacerbate risk^(^^13^^)^.

The limitations of the present study are that it was a cross-sectional study, that the 3 d diet diary may not have been long enough to be representative of intake, and the women may have under-reported. Women may have exercised more and/or eaten less because of awareness of the recording procedures. There is some evidence that energy expenditure is lower in postmenopausal women during exercise as discussed above, and since menopausal status is not considered in the algorithms used for the accelerometer outputs; this could have resulted in over-estimation of the energy expenditure in the postmenopausal women, making the difference between the groups even greater. The postmenopausal women were older than the premenopausal women but this allowed us to exclude women in the peri-menopausal period, giving two well-defined groups. We had objective measures of activity and fatty acid intake and a novel, thorough combination of dietary and anthropometric measurements in groups well matched for waist and BMI. The design of the study means that there is a possibility of recruitment of more health-conscious individuals, especially in the postmenopausal group. Despite this issue, plasma LDL and blood pressure were significantly higher, and BMD was significantly lower in the postmenopausal women. Given the difference in energy intake and expenditure found in the study, it would have been interesting to measure plasma leptin concentrations.

The importance of maintaining body weight in mid-life cannot be underestimated for women where age, menopausal status and BMI are risk factors for CVD. We have shown that an equal BMI and waist circumference in post- compared with premenopausal women is associated with a significantly lower TEE and a tendency for lower energy intake. However, the maintenance of BMI in this way has the potential for compromised bone health in postmenopausal women; postmenopausal women spent less time performing moderate exercise and this was associated with a lower BMD. However, the main lifestyle determinant of BMD in postmenopausal women was dietary fatty acid composition (lower dietary *n*-6:*n*-3 ratio) and for premenopausal women, TEE predicted BMD. In summary, although the avoidance of weight gain in mid-life is beneficial for women's health, it should not be at the expense of reduced exercise, or diet quality, in order to help prevent the decline in bone health, and increasing CVD risk.

## References

[1] Heller RF & Jacobs HS (1978) Coronary heart disease in relation to age, sex, and the menopause. BMJ 1, 472–474 626838 10.1136/bmj.1.6111.472 PMC1603172

[2] Riggs BL, Khosla S & Melton LJ III (2002) Sex steroids and the construction and conservation of the adult skeleton. Endocr Rev 23, 279–302 12050121 10.1210/edrv.23.3.0465

[3] Marcovitz PA, Tran HH, Franklin BA, (2005) Usefulness of bone mineral density to predict significant coronary artery disease. Am J Cardiol 96, 1059–1063 16214438 10.1016/j.amjcard.2005.06.034

[4] Wardlaw GM (1996) Putting body weight and osteoporosis into perspective. Am J Clin Nutr 63, 433S–436S 8615336 10.1093/ajcn/63.3.433

[5] Yang S, Nguyen ND, Center JR, (2013) Association between abdominal obesity and fracture risk: a prospective study. J Clin Endocrinol Metab 98, 2478–2483 23559081 10.1210/jc.2012-2958

[6] Hagen KB, Dagfinrud H, Moe RH, (2012) Exercise therapy for bone and muscle health: an overview of systematic reviews. BMC Med 10, 167. 10.1186/1741-7015-10-167 PMC3568719 23253613

[7] Lovejoy JC, Champagne CM, de Jonge L, (2008) Increased visceral fat and decreased energy expenditure during the menopausal transition. Int J Obes (Lond) 32, 949–958 18332882 10.1038/ijo.2008.25 PMC2748330

[8] Craig R & Mindell J (editors) (2012) Health Survey for England 2011: Health, Social Care and Lifestyles. Leeds: Health and Social Care Information Centre

[9] Sowers M, Zheng H, Tomey K, (2007) Changes in body composition in women over six years at midlife: ovarian and chronological aging. J Clin Endocrinol Metab 92, 895–901 17192296 10.1210/jc.2006-1393 PMC2714766

[10] Wing RR, Matthews KA, Kuller LH, (1991) Weight gain at the time of menopause. Arch Intern Med 151, 97–102 1985614

[11] Liu A, Abbasi F & Reaven GM (2011) Adiposity indices in the prediction of metabolic abnormalities associated with cardiovascular disease in non-diabetic adults. Nutr Metab Cardiovasc Dis 21, 553–560 20304617 10.1016/j.numecd.2009.12.009 PMC2895680

[12] Matthews KA, Crawford SL, Chae CU, (2009) Are changes in cardiovascular disease risk factors in midlife women due to chronological aging or to the menopausal transition? J Am Coll Cardiol 54, 2366–2373 20082925 10.1016/j.jacc.2009.10.009 PMC2856606

[13] Williams PT & Thompson PD (2013) Walking *versus* running for hypertension, cholesterol, and diabetes mellitus risk reduction. Arterioscler Thromb Vasc Biol 33, 1085–1091 23559628 10.1161/ATVBAHA.112.300878 PMC4067492

[14] Wang Q, Hassager C, Ravn P, (1994) Total and regional body-composition changes in early postmenopausal women: age-related or menopause-related? Am J Clin Nutr 60, 843–848 7985622 10.1093/ajcn/60.6.843

[15] Cunningham JJ (1982) Body composition and resting metabolic rate: the myth of feminine metabolism. Am J Clin Nutr 36, 721–726 7124675 10.1093/ajcn/36.4.721

[16] Abildgaard J, Pedersen AT, Green CJ, (2013) Menopause is associated with decreased whole body fat oxidation during exercise. Am J Physiol Endocrinol Metab 304, E1227–E1236 23548615 10.1152/ajpendo.00492.2012

[17] Macdonald HM, New SA & Reid DM (2005) Longitudinal changes in dietary intake in Scottish women around the menopause: changes in dietary pattern result in minor changes in nutrient intake. Public Health Nutr 8, 409–416 15975187 10.1079/phn2005705

[18] Alberti KG, Zimmet P & Shaw J (2005) The metabolic syndrome – a new worldwide definition. Lancet 366, 1059–1062 16182882 10.1016/S0140-6736(05)67402-8

[19] British Heart Foundation (2013) Preventing Heart Disease. http://www.bhf.org.uk/heart-health/preventing-heart-disease/your-weight.aspx (accessed May 2013).

[20] Tan GD, Neville MJ, Liverani E, (2006) The *in vivo* effects of the Pro12Ala PPARγ2 polymorphism on adipose tissue NEFA metabolism: the first use of the Oxford Biobank. Diabetologia 49, 158–168 16362285 10.1007/s00125-005-0044-z

[21] Bingham SA, Welch AA, McTaggart A, (2001) Nutritional methods in the European Prospective Investigation of Cancer in Norfolk. Public Health Nutr 4, 847–858 11415493 10.1079/phn2000102

[22] McQuaid SE, Hodson L, Neville MJ, (2011) Downregulation of adipose tissue fatty acid trafficking in obesity: a driver for ectopic fat deposition? Diabetes 60, 47–55 20943748 10.2337/db10-0867 PMC3012196

[23] Hodson L, Neville M, Chong MF, (2013) Micro-techniques for analysis of human adipose tissue fatty acid composition in dietary studies. Nutr Metab Cardiovasc Dis 23, 1128–1133 23228218 10.1016/j.numecd.2012.11.003

[24] National Cholesterol Education Program (NCEP) Expert Panel on Detection, Evaluation, and Treatment of High Blood Cholesterol in Adults (Adult Treatment Panel III) (2002) Third Report of the National Cholesterol Education Program (NCEP) Expert Panel on Detection, Evaluation, and Treatment of High Blood Cholesterol in Adults (Adult Treatment Panel III) final report. Circulation 106, 3143–3421 12485966

[25] Hodson L, Skeaff CM & Fielding BA (2008) Fatty acid composition of adipose tissue and blood in humans and its use as a biomarker of dietary intake. Prog Lipid Res 47, 348–380 18435934 10.1016/j.plipres.2008.03.003

[26] Aslibekyan S, Campos H & Baylin A (2012) Biomarkers of dairy intake and the risk of heart disease. Nutr Metab Cardiovasc Dis 22, 1039–1045 21549582 10.1016/j.numecd.2011.02.003 PMC3595059

[27] Cao Y, Zhang S, Zou S, (2013) The relationship between endogenous androgens and body fat distribution in early and late postmenopausal women. PLOS ONE 8, e58448. 10.1371/journal.pone.0058448 PMC3587576 23484029

[28] Clark JM, Brancati FL & Diehl AM (2002) Nonalcoholic fatty liver disease. Gastroenterology 122, 1649–1657 12016429 10.1053/gast.2002.33573

[29] Volzke H, Schwarz S, Baumeister SE, (2007) Menopausal status and hepatic steatosis in a general female population. Gut 56, 594–595 17369390 10.1136/gut.2006.115345 PMC1856852

[30] Brown WJ, Williams L, Ford JH, (2005) Identifying the energy gap: magnitude and determinants of 5-year weight gain in midage women. Obes Res 13, 1431–1441 16129726 10.1038/oby.2005.173

[31] Gibney MJ, Vorster HH & Kok FJ (editors) (2002) Introduction to Human Nutrition. Oxford: Blackwell Science Ltd

[32] Goldberg GR, Black AE, Jebb SA, (1991) Critical evaluation of energy intake data using fundamental principles of energy physiology: 1. Derivation of cut-off limits to identify under-recording. Eur J Clin Nutr 45, 569–581 1810719

[33] Goris AH & Westerterp KR (1999) Underreporting of habitual food intake is explained by undereating in highly motivated lean women. J Nutr 129, 878–882 10203564 10.1093/jn/129.4.878

[34] Goris AH, Westerterp-Plantenga MS & Westerterp KR (2000) Undereating and underrecording of habitual food intake in obese men: selective underreporting of fat intake. Am J Clin Nutr 71, 130–134 10617957 10.1093/ajcn/71.1.130

[35] Kristiansen J, Korshoj M, Skotte JH, (2011) Comparison of two systems for long-term heart rate variability monitoring in free-living conditions – a pilot study. Biomed Eng Online 10, 27. 10.1186/1475-925X-10-27 PMC3080340 21481282

[36] Villars C, Bergouignan A, Dugas J, (2012) Validity of combining heart rate and uniaxial acceleration to measure free-living physical activity energy expenditure in young men. J Appl Physiol 113, 1763–1771 23019315 10.1152/japplphysiol.01413.2011

[37] Loney T, Standage M, Thompson D, (2011) Self-report *vs.* objectively assessed physical activity: which is right for public health? J Phys Act Health 8, 62–70 21297186 10.1123/jpah.8.1.62

[38] Cancer Research UK (2013) Physical activity recommendations. http://www.cancerresearchuk.org/cancer-info/cancerstats/causes/lifestyle/physicalactivity/physical-activity-and-risk-of-cancer#Physical3 (accessed 12 May 2013).

[39] Nielsen MS, Schmidt EB, Stegger J, (2013) Adipose tissue arachidonic acid content is associated with the risk of myocardial infarction: a Danish case–cohort study. Atherosclerosis 227, 386–390 23390891 10.1016/j.atherosclerosis.2012.12.035

[40] Scientific Advisory Committee on Nutrition (2004) Advice on Fish Consumption: Benefits and Risks. Norwich: The Stationery Office

[41] Kruger MC, Coetzee M, Haag M, (2010) Long-chain polyunsaturated fatty acids: selected mechanisms of action on bone. Prog Lipid Res 49, 438–449 20600307 10.1016/j.plipres.2010.06.002

[42] Albertazzi P & Coupland K (2002) Polyunsaturated fatty acids. Is there a role in postmenopausal osteoporosis prevention? Maturitas 42, 13–22 12020975 10.1016/s0378-5122(02)00022-1

[43] Kruger MC, Coetzer H, de Winter R, (1998) Calcium, γ-linolenic acid and eicosapentaenoic acid supplementation in senile osteoporosis. Aging (Milano) 10, 385–394 9932142 10.1007/BF03339885

[44] Bassey EJ, Littlewood JJ, Rothwell MC, (2000) Lack of effect of supplementation with essential fatty acids on bone mineral density in healthy pre- and postmenopausal women: two randomized controlled trials of Efacal *v.* calcium alone. Br J Nutr 83, 629–635 10911771 10.1017/s0007114500000805

[45] Hodson L, Skeaff CM & McKenzie JE (2002) Maximal response to a plasma cholesterol-lowering diet is achieved within two weeks. Nutr Metab Cardiovasc Dis 12, 291–295 12616809

[46] Schmitz G & Ecker J (2008) The opposing effects of *n*-3 and *n*-6 fatty acids. Prog Lipid Res 47, 147–155 18198131 10.1016/j.plipres.2007.12.004

[47] Salamone LM, Glynn N, Black D, (1995) Body composition and bone mineral density in premenopausal and early perimenopausal women. J Bone Miner Res 10, 1762–1768 8592954 10.1002/jbmr.5650101120

[48] Sowers M, Kshirsagar A, Crutchfield M, (1991) Body composition, age and femoral bone mass of young adult women. Ann Epidemiol 1, 245–254 1669505 10.1016/1047-2797(91)90003-u

[49] Reid IR, Ames R, Evans MC, (1992) Determinants of total body and regional bone mineral density in normal postmenopausal women – a key role for fat mass. J Clin Endocrinol Metab 75, 45–51 1619030 10.1210/jcem.75.1.1619030

[50] Nguyen TV, Howard GM, Kelly PJ, (1998) Bone mass, lean mass, and fat mass: same genes or same environments? Am J Epidemiol 147, 3–16 9440393 10.1093/oxfordjournals.aje.a009362

[51] Arimatsu M, Kitano T, Kitano N, (2009) Correlation between bone mineral density and body composition in Japanese females aged 18–40 years with low forearm bone mineral density. Environ Health Prev Med 14, 46–51 19568867 10.1007/s12199-008-0056-7 PMC2684765

[52] Moseley KF, Dobrosielski DA, Stewart KJ, (2011) Lean mass and fat mass predict bone mineral density in middle-aged individuals with noninsulin-requiring type 2 diabetes mellitus. Clin Endocrinol (Oxf) 74, 565–571 21198741 10.1111/j.1365-2265.2010.03965.x

[53] Douchi T, Kuwahata R, Matsuo T, (2003) Relative contribution of lean and fat mass component to bone mineral density in males. J Bone Miner Metab 21, 17–21 12491089 10.1007/s007740300003

[54] Arimatsu M, Kitano T, Kitano N, (2005) Correlation between forearm bone mineral density and body composition in Japanese females aged 18–40 years. Environ Health Prev Med 10, 144–149 21432153 10.1007/BF02900807 PMC2723254

[55] Hsu YH, Venners SA, Terwedow HA, (2006) Relation of body composition, fat mass, and serum lipids to osteoporotic fractures and bone mineral density in Chinese men and women. Am J Clin Nutr 83, 146–154 16400063 10.1093/ajcn/83.1.146

[56] Douchi T, Oki T, Nakamura S, (1997) The effect of body composition on bone density in pre- and postmenopausal women. Maturitas 27, 55–60 9158078 10.1016/s0378-5122(97)01112-2

[57] Reid IR, Legge M, Stapleton JP, (1995) Regular exercise dissociates fat mass and bone density in premenopausal women. J Clin Endocrinol Metab 80, 1764–1768 7775619 10.1210/jcem.80.6.7775619

[58] NHS (2013) Vitamins and minerals – calcium. http://www.nhs.uk/Conditions/vitamins-minerals/Pages/Calcium.aspx (accessed 7 June 2013).

[59] British Dietetic Association (2013) Calcium. http://www.bda.uk.com/foodfacts/Calcium.pdf (accessed 12 May 2013).

[60] World Health Organization & Food and Agriculture Organization of the United Nations (2004) *Vitamin and Mineral Requirements in Human Nutrition*, 2nd ed. Report of a Joint FAO/WHO Expert Consultation on Human Vitamin and Mineral Requirements, Bangkok, Thailand, 21–30 September 1998. Geneva: WHO.

